# Large-Scale Model Test on Water Pressure Resistance of Lining Structure of Water-Rich Tunnel

**DOI:** 10.3390/ma16010440

**Published:** 2023-01-03

**Authors:** Mingli Huang, Meng Huang, Ze Yang

**Affiliations:** 1 Key Laboratory for Urban Underground Engineering of the Education Ministry, Beijing Jiaotong University, Beijing 100044, China; 2 School of Civil Engineering, Beijing Jiaotong University, Beijing 100044, China

**Keywords:** water-rich tunnel, lining structure, water pressure resistance, large-scale model test, numerical calculation

## Abstract

In order to solve the problem of testing the water pressure resistance of lining structures of water-rich tunnels and the difficulty of implementing the existing model tests, a large-scale model test method was proposed relying on the New Yuanliangshan Tunnel threatened by high pressure and rich water. This method creatively transformed the external water pressure of the lining structure into internal water pressure, and the conversion coefficient of water resistance of lining under different sizes and loading modes was obtained by numerical calculation. Results showed that the ultimate water pressure resistance of the lining structure under an external uniformly distributed water pressure and local water pressure was 1.44 and 0.67 times of that obtained from the large-scale model tests, respectively. By conducting the large-scale model tests and combining with the conversion coefficient, the water pressure resistance of the actual tunnel lining could be obtained. Research indicated that water pressure resistance of K2.0 (bearing water pressure of 2.0 MPa) type lining at the transition section of karst caves and K3.0 (bearing water pressure of 3.0 MPa) type lining at the section of karst caves of the New Yuanliangshan Tunnel was 3.33 MPa and not less than 4.36 MPa, respectively, and the high reliability of the large-scale model tests was verified by numerical calculation, implying that the model test method could be extended to similar tunnel projects.

## 1. Introduction

China has a vast territory, and more than two-thirds of which are mountains and hills, especially in the western region. With the planning and rapid implementation of the traffic network in western China, the number of tunnels has also dramatically increased. However, water-rich geology is common in western China, and more and more tunnels that have been built, are under construction or under planning, will inevitably pass through water-rich areas. Under the action of high water pressure, tunnel lining may suffer from leakage, cracking, etc., and even engineering disasters such as water and mud inrush, which seriously threaten the safety of construction and operation of tunnels. Therefore, it is of great significance to study the ultimate water pressure resistance of tunnel lining structure to provide a reference for lining design and the construction of high-pressure and water-rich tunnels.

Many scholars have carried out a series of related studies on the stress law of tunnel lining structure under water pressure, and achieved fruitful results. Wang et al. [1] studied the distribution of water pressure on tunnel lining by theoretical analysis, indoor test and field measurement, and the research indicated that the grouting zone cannot reduce water pressure on lining with complete waterproofing. Only when drainage measures were taken could the grouting zone be effective in reducing water pressure on lining. Ding et al. [2] found that there is obvious stress concentration and mutation at the side wall, invert corner and invert of the tunnel lining under external water pressure, which greatly reduced the safety. Shin et al. [3] established the seepage field model of surrounding rock-tunnel under drainage and undrained boundary conditions by using the numerical calculation method, and revealed the distribution law of water pressure behind the lining under different working conditions. Bian et al. [4] revealed the cracking law of hydraulic tunnel lining under high internal water pressure based on elastic damage theory and linear elastic fracture mechanics theory. Li et al. [5] obtained the calculation formula of water pressure characteristics of tunnel lining through theoretical formula derivation, and revealed the water pressure characteristics of lining of mountain tunnel with high water pressure. Kentaro et al. [6] and Huang et al. [7] studied the distribution characteristics of external water pressure of lining structure of karst tunnel with high water pressure, and summarized the water pressure characteristics of tunnel lining under the condition of water blocking and drainage restriction. Xin et al. [8] analyzed the mechanical characteristics of tunnel supports and linings with respect to seepage and pressure by using numerical method and a test model based on the tunnel mechanics and seepage mechanics. Ren et al. [9] and Khezri et al. [10] carried out relevant researches on influencing factors and values of external water pressure of tunnel lining. Shen [11] analyzed the water pressure characteristics of the secondary lining of karst tunnels by means of model test, theoretical analysis and on-site monitoring, and proposed a generalized model for calculating water pressure of karst tunnel lining. He et al. [12] deduced a nonlinear analytical formula for water load of support system of mountain tunnel in water-rich area based on the limited drainage of blind pipe and the water separation effect of waterproof board. Fang et al. [13] carried out the indoor loading model test on the stress characteristics of lining structure of large section highway tunnel under external water pressure based on the composite simulation test platform of tunnel-stratum. Ma et al. [14] obtained the universal solution of Laplace equation in the conformal variation circle area, and deduced the analytical solution of water pressure of tunnel lining with steady seepage according to boundary condition and seepage continuity. Wan et al. [15] studied the stress characteristics and safety of lining structure of single-track railway tunnel under local water pressure by means of field test and numerical simulation, and proposed the design parameters of tunnel lining under local water pressure. Wang et al. [16] analyzed the characteristics of external water pressure behind a secondary lining of tunnel via numerical method. Wang et al. [17] studied the stress characteristics, structural safety and failure process of tunnel lining under the combined action of surrounding rock pressure and external water pressure by using model test and numerical calculation methods. Liu et al. [18] took the Tongxi karst tunnel as an example to study the impact of incremental changes in external water pressure on tunnel lining structure. Huang et al. [19] considered that the water pressure on the lining (WPOL) has a significant impact on the parameter selection and operation safety of the lining for mountain tunnels in water-rich areas, and derived the analytical expressions of the WPOL based on the theory of groundwater dynamics and complex function. Zhang et al. [20] utilized the analytical calculation method to study the influence of the change of tunnel seepage and water pressure on the water pressure behind the lining during the construction process based on the New Yuanliangshan Tunnel. Yuan et al. [21] carried out research on a prediction model of water pressure behind the lining structure of a tunnel with high water pressure. Jin et al. [22] established the internal force calculation model under the combined action of local high water pressure at the top of circular lining section and surrounding rock pressure by using theoretical analysis method. Fan et al. [23,24] and Bao et al. [25] indicated that, with the frequent occurrence of heavy rainfall, the lining cracking, leakage, and collapse accidents of water-rich mountain tunnels were becoming increasingly prominent. Ding et al. [26] studied the water pressure change of exposing existing karst caves in the tunnel construction, and used double-layer primary support to meet the construction safety based on the Yongfutun Tunnel. Zhao et al. [27] discussed the lining water pressure and its reduction coefficient of the horseshoe section tunnel, and studied the stress law of tunnel lining under different water reduction coefficients. Huang et al. [28] studied the water pressure resistance of composite structure of grouting stone body and lining of karst tunnel with high water pressure by means of numerical calculation. Additionally, Rosso et al. [29] proposed an indirect and non-destructive testing technology based on artificial intelligence (AI), which can be used to evaluate the states of tunnel linings and indirectly assess the water pressure acting on tunnel linings.

In summary, the existing research on water pressure resistance of lining structures, including model tests and numerical simulation, were generally carried out by using the loading mode of external water pressure on the tunnel lining, applying water head on the outer boundary of the model or setting seepage boundary conditions to make the water pressure finally act on the outer surface of the tunnel lining. For the model test, the method of applying external water pressure on the outer surface of tunnel lining is more consistent with the actual water pressure acting on lining structure, but it is generally difficult to carry out. However, the large-scale model test carried out in this paper adopted the method of water pressure loading on the inner surface of the lining. Additionally, the numerical calculation method adopted in this paper was mainly to obtain the maximum water pressure resistance of lining structure by analyzing the failure process of tunnel lining under high water pressure, which was different from the focus of existing research.

At present, the test and verification of the water pressure resistance of lining structures of high-pressure and water-rich tunnels is still very challenging, and the existing model tests using the loading mode of external water pressure are relatively difficult to implement. To solve this problem, relying on the New Yuanliangshan Tunnel project, a large-scale model test method for testing and verifying the ultimate water pressure resistance of lining structure of water-rich tunnel was designed and carried out in this paper, and the reliability of the model test method was verified by utilizing numerical calculation. The main organization of this paper is as follows: Section 1 contains the “Introduction”; Section 2 presents the materials and methods of the large-scale model tests and numerical calculation; Section 3 discusses the results of large-scale model tests and numerical calculation; Section 4 contains the “Conclusions”, which condensed the research results. The results of this research were intended to provide reference for the design and construction of water-resistant lining of water-rich tunnels.

## 2. Materials and Methods

### 2.1. Test Materials and Mix Proportion

The concrete mixtures used in the large-scale model test include Portland cement, potable water, coarse aggregates, fine aggregates and admixture. The material properties of each mixture were shown in Table 1. Physical and mechanical properties of Portland cement were listed in Table 2. Potable water was selected, quality of which was shown in Table 3, and the water-cement ratio was 0.53. Considering the potable water contains a very small amount of impurities, it has no bad effect on the performance of concrete, so the negative influence of water quality can be excluded when conducting the model tests on water pressure resistance of tunnel lining. The mix ratios of concrete were listed in Table 4.

### 2.2. Experimental Procedure

#### 2.2.1. Engineering Background

**Figure 1:**
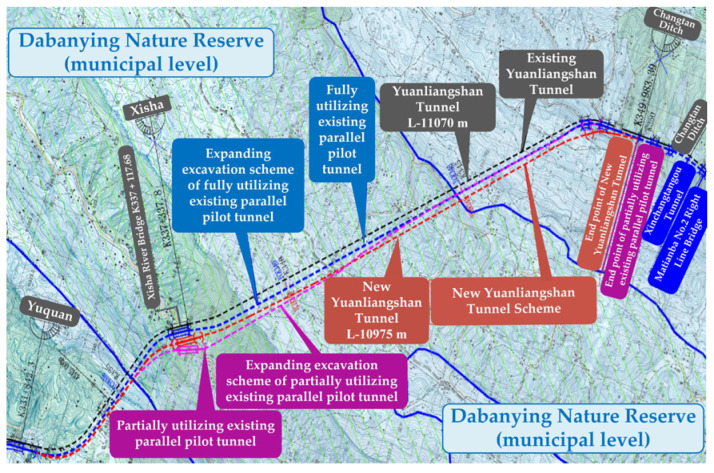
Location map of the New Yuanliangshan Tunnel.

The New Yuanliangshan Tunnel has a total length of 11.077 km, which is constructed by fully utilizing the parallel pilot of the existing Yuanliangshan Tunnel for expanded excavation. The location map of the New Yuanliangshan Tunnel is shown in Figure 1. Lining structure of the New Yuanliangshan Tunnel is described as follows:

##### Primary Lining

The waterproof capacity of primary lining is limited, but the construction of primary lining is essential to ensure construction safety. CF25 steel fiber shotcrete was used for the primary lining of the New Yuanliangshan Tunnel. The thickness of shotcrete was determined according to the level of surrounding rock and location. The thickness of shotcrete in the section of karst cave and transition section of karst cave was 20 cm. Φ22 and Φ25 combined hollow grouting anchor bars were adopted in the tunnel arch of surrounding rocks at all levels, and Φ22 and Φ32 mortar anchor bars were set in the side wall. The reinforcement mesh was made of HPB300 steel bar with a diameter of 6.5~12 mm. The small conduit was a kind of steel flower tube with a diameter of 42 mm and thickness of 3.5 mm. I16, I18, I20, I25b and H200 × 150 were adopted for the steel frame of primary lining.

##### Secondary Lining

**Figure 2:**
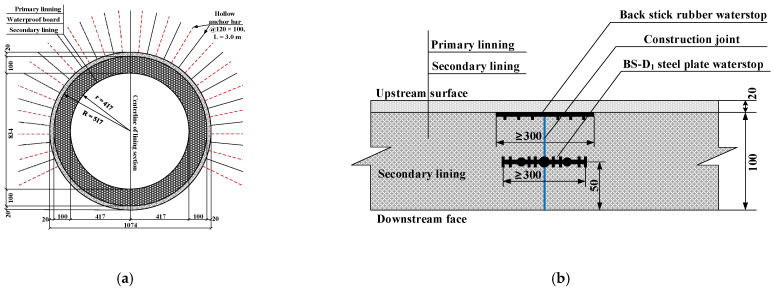
Structural drawing of secondary lining in the transition section of karst cave (unit: cm): (**a**) K2.0 type lining; (**b**) Type II construction joint (circumferential).

The construction quality of secondary lining structure can be basically guaranteed, so the leakage of tunnel lining is generally easy to occur at the construction joints of tunnel lining. The maximum design value of water pressure of lining structure of the New Yuanliangshan Tunnel under the extreme condition of water blocking and drainage restriction was up to 3.0 MPa, which put forward high requirements for design and construction of tunnel lining. For the high-pressure and water-rich area of the New Yuanliangshan Tunnel, karst is very developed. The surrounding rock at all levels in the transition section of the karst cave was provided with the circular reinforced concrete lining (K2.0 type) bearing water pressure of 2.0 MPa, with a lining thickness of 100 cm. The type II construction joint was selected as the circumferential construction joint, and the arrangement of waterstop was a combination of rubber waterstop and mid-buried steel plate waterstop. The cross section and circumferential construction joint of the lining structure in the transition section of the karst cave were shown in Figure 2.

**Figure 3:**
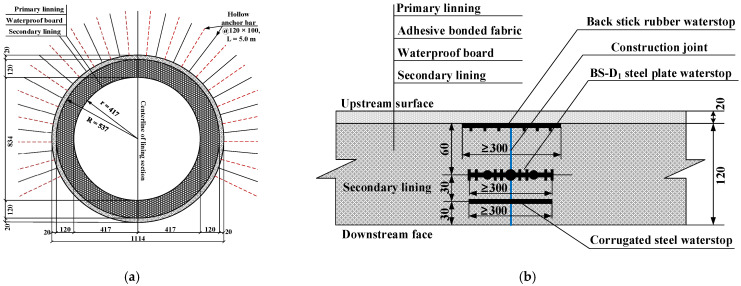
Structural drawing of secondary lining in the section of karst cave (unit: cm): (**a**) K3.0 type lining; (**b**) type III construction joint (circumferential).

For the karst cave section in the high-pressure and water-rich area, the circular reinforced concrete lining (K3.0 type) capable of withstanding water pressure of 3.0 MPa was set, with a lining thickness of 120 cm. The type III construction joint was adopted as the circumferential construction joint, and the arrangement of waterstop was a combination of back stick rubber waterstop, mid-buried steel plate waterstop and mid-buried corrugated steel waterstop. The cross section and circumferential construction joint of lining structure in the section of the karst cave were shown in Figure 3.

#### 2.2.2. Large Scale Model Test

##### Model Test Principle

According to the distribution characteristics of tangential and radial stresses of ring or cylinder subjected to uniform internal and external pressure in the elasticity theory, we know that under the action of external uniform water pressure, the stress of radial and tangential is compressive stress. However, under the action of internal uniform pressure, the radial stress is compressive stress and the tangential stress is tensile stress. As we all know, the compressive strength of tunnel lining concrete is far greater than the tensile strength. Therefore, the tunnel lining is subject to tensile failure under the action of internal water pressure and compressive shear failure under the action of external water pressure. Actually, tunnel lining bears the effect of external water pressure, but it is difficult to carry out large-scale model test of water pressure resistance of tunnel lining by means of external water pressure loading. Therefore, from the point of view of the ultimate water pressure that tunnel lining can withstand when it is finally destroyed, a method of loading water pressure inside the model was proposed to transform the problem of tunnel lining bearing external water pressure into that of bearing internal water pressure for research in this paper, and the conversion relationship between the safety factor of water pressure resistance under the action of external water pressure and internal water pressure was established. According to the water pressure resistance of tunnel lining under internal water pressure obtained from indoor model test and the conversion coefficient obtained from numerical calculation, the water pressure resistance of lining under external water pressure can be obtained through reverse calculation.

**Figure 4:**
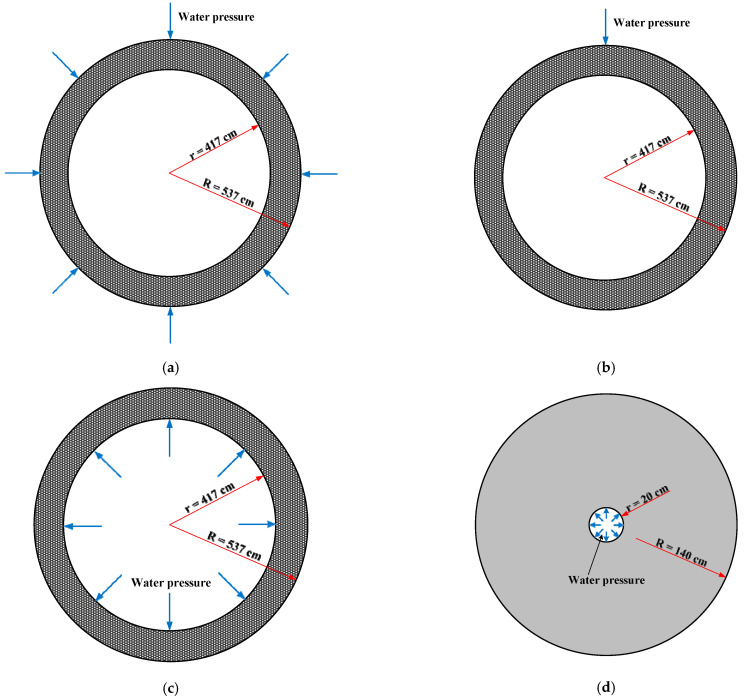
Schematic diagram of numerical calculation model (unit: cm): (**a**) I: lining bearing external uniform water pressure; (**b**) II: lining bearing external local water pressure; (**c**) III: lining bearing internal uniform water pressure; (**d**) IV: large-scale test model bearing internal uniform water pressure.

The inner diameter of lining structure of the New Yuanliangshan Tunnel was 8 m. In order to prepare the large-scale models, the inner diameter of the models was reduced to 0.4 m on the basis of ensuring that the thickness of the lining and the form of the construction joint were unchanged. The conversion relationship of safety factor between water pressure resistance of the test model and the lining was established by numerical calculation. Safety factor referred to the ratio of the actual water pressure resistance of tunnel lining to the design value of water pressure resistance. The higher the safety factor, the stronger the water pressure resistance of tunnel lining. The water pressure around the karst tunnel that deep buried was considered as the uniform water pressure and was replaced by a uniform load. Numerical calculation software of RFPA (Realistic Failure Process Analysis) was adopted to analyze the conversion relationship of safety factors of water pressure resistance of tunnel lining. RFPA is a numerical analysis software of material fracture process based on finite element stress analysis and statistical damage theory, which can simulate the whole process of the material from progressive failure to instability. There were four numerical calculation conditions in total, as shown in Table 5. The numerical calculation models were shown in Figure 4. The micro mechanical parameters of C30 concrete were selected as the material of numerical calculation models, as shown in Table 6.

(1) Numerical calculation results under condition I

**Figure 5:**
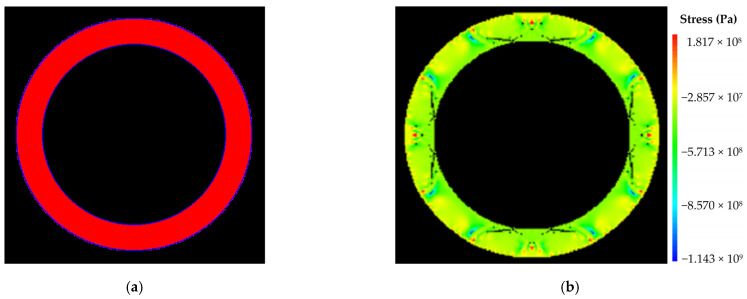
Numerical calculation results under condition I: (**a**) calculation model; (**b**) damage diagram of the model (step: 91–6).

The size of numerical calculation model was 12 m × 12 m, with an internal radius of 4.17 m. The number of units was 400 × 400, with a unit size of 30 mm × 30 mm. The initial value of water pressure was 0 MPa, and the single step increment was 0.1 MPa. The numerical calculation models and results were shown in Figure 5. As shown in Figure 5b, the model was damaged when it was loaded to 91 steps, and the external pressure of the model was 9.1 MPa, so the ultimate water pressure resistance of the lining structure under external uniform water pressure was 9.1 MPa.

(2) Numerical calculation results under condition II

**Figure 6:**
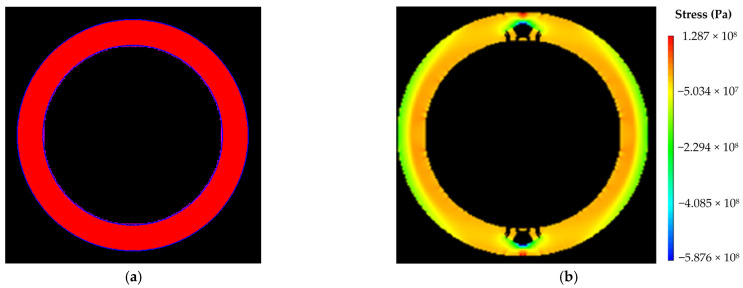
Numerical calculation results under condition II: (**a**) calculation model; (**b**) damage diagram of the model (step: 42–5).

The size of numerical calculation model was 12 m × 12 m, with an internal radius of 4.17 m. The number of units was 400 × 400, with a unit size of 30 mm × 30 mm. The initial value of water pressure was 0 MPa, and the single step increment was 0.1 MPa. The numerical calculation models and results were shown in Figure 6. As shown in Figure 6b, the model was damaged when it was loaded to 42 steps, and the external local pressure of the model was 4.2 MPa, so the ultimate water pressure resistance of the lining structure under external local water pressure was 4.2 MPa.

(3) Numerical calculation results under condition III

**Figure 7:**
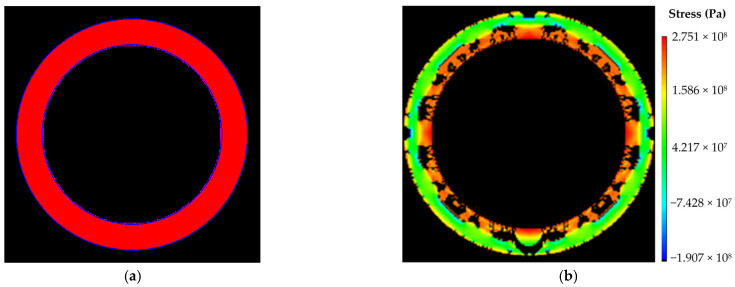
Numerical calculation results under condition III: (**a**) calculation model; (**b**) damage diagram of the model (step: 30–5).

The size of numerical calculation model was 12 m × 12 m, with an internal radius of 4.17 m. The number of units was 400 × 400, with a unit size of 30 mm × 30 mm. The initial value of water pressure was 0 MPa, and the single step increment was 0.1 MPa. The numerical calculation models and results were shown in Figure 7. As shown in Figure 7b, the model was damaged when it was loaded to 30 steps, and the internal pressure of the model was 3.0 MPa, so the ultimate water pressure resistance of the lining structure under internal uniform water pressure was 3.0 MPa.

(4) Numerical calculation results under condition IV

**Figure 8:**
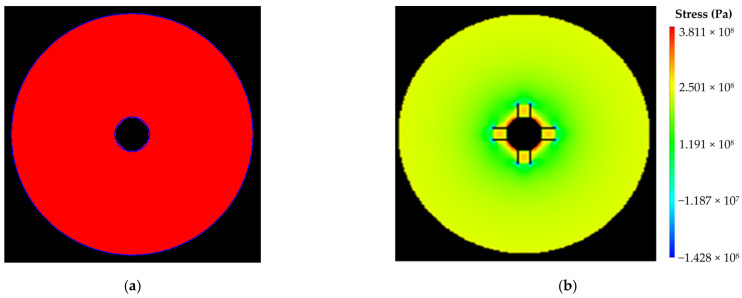
Numerical calculation results under condition IV: (**a**) calculation model; (**b**) damage diagram of the model (step: 63–14).

The size of numerical calculation model was 3 m × 3 m, with an internal radius of 0.2 m. The number of units was 300 × 300, with a unit size of 10 mm × 10 mm. The initial value of water pressure was 0 MPa, and the single step increment was 0.1 MPa. The numerical calculation models and results were shown in Figure 8. As shown in Figure 8b, the model was damaged when it was loaded to 63 steps, and the internal pressure of the model was 6.3 MPa, so the ultimate water pressure resistance of the large-scale test model under internal uniform water pressure was 6.3 MPa.

(5) Analysis of numerical calculation results

**Figure 9:**
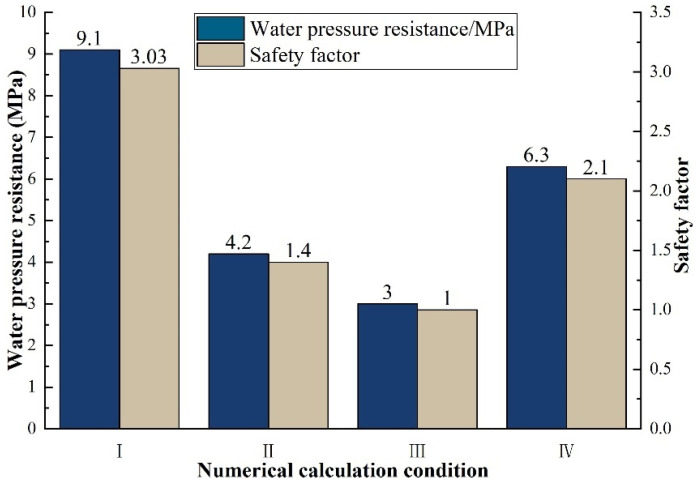
Water pressure resistance and safety factor under different working conditions.

The calculation results of safety factor of water pressure resistance were shown in Table 7. As shown in Figure 9, bar chart of changing of water pressure resistance and safety factor under different working conditions was drawn.

It can be seen from Table 7 and Figure 9 that the water pressure resistance of tunnel lining under numerical calculation condition I was the largest (9.1 MPa), while that under condition III is the smallest (3.0 MPa), and the calculation condition Ⅳ was the simulation of large-scale model test of water pressure resistance of tunnel lining. In order to facilitate the analysis, the normalization was carried out based on 3.0 MPa. Results showed that the ultimate water pressure resistance of the lining of condition I under uniform external water pressure was 1.44 times that of the large-scale model test of condition IV under uniform internal water pressure, while the ultimate water pressure resistance of the lining of condition II under local external water pressure was 0.67 times that of condition IV. In other words, the ultimate water pressure resistance of tunnel lining structure under external uniform water pressure and external local water pressure was 1.44 times and 0.67 times of that of tunnel lining obtained from large-scale model test under uniform internal water pressure, respectively.

##### Preparation of Specimens

**Figure 10:**
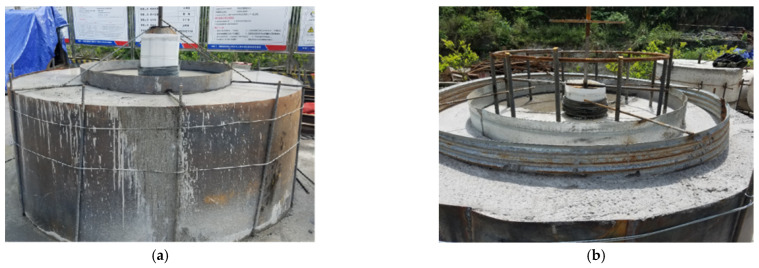
Large scale test models: (**a**) specimen 1; (**b**) specimen 2.

**Figure 11:**
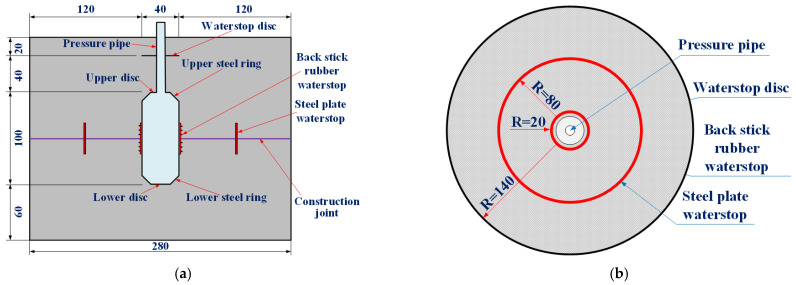
Schematic diagram and dimensions of the model for testing water pressure of lining in the transition section of karst cave (unit: cm): (**a**) elevation view of model; (**b**) vertical view of model.

**Figure 12:**
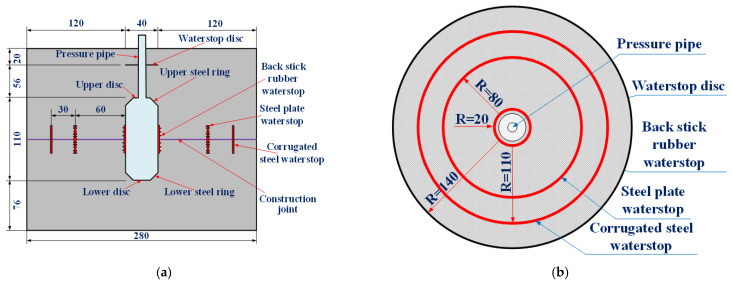
Schematic diagram and dimensions of the model for testing water pressure of lining in the section of karst cave (unit: cm): (**a**) elevation view of model; (**b**) vertical view of model.

In order to test the water pressure resistance of the lining structure (K2.0 type) in the transition section of karst cave and the lining structure (K3.0 type) in the section of karst cave, two test models under two different conditions were prepared, respectively, and their use conditions and waterstop settings were shown in Table 8. The preparation process of the test models was as followed. Steel molds were made at first, and then the concrete was mixed according to the mixing ratio in Table 4. Each of the two test models were divided into upper and lower parts for pouring, and the interface between the upper and lower parts of each model was set to simulate the circumferential construction joint of tunnel lining. The waterstop and pressure pipe were fixed at the specified position. After the lower part of each test model was poured and maintained to sufficient strength, the upper part was subsequently poured and maintained. Finally, the test models were obtained. The maintenance of test models was complied with Technical Guide for Construction of Railway Tunnel Engineering (TZ 204-2008). The test models that completely poured and maintained were shown in Figure 10. The schematic diagram and dimensions of the two test models were shown in Figure 11 and Figure 12.

##### Design of Pressurization System

**Figure 13:**
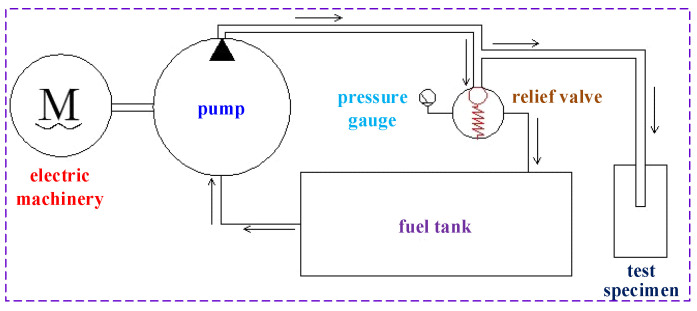
Schematic diagram of pressure pump station.

**Figure 14:**
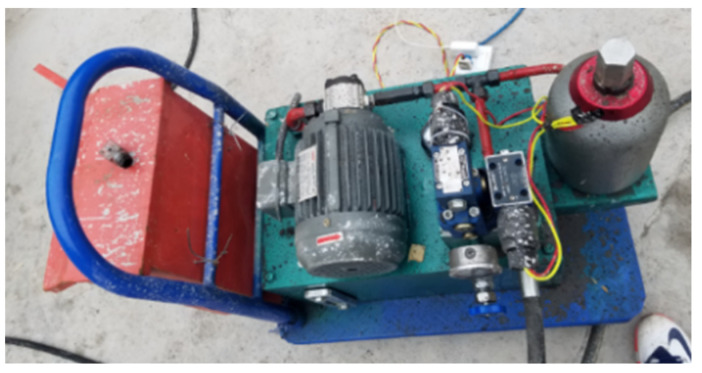
Pressure pump used on site.

The pressure pump station was used to pressurize the system, which was composed of a power system, a distribution system and a working system. The power system was mainly composed of a motor and a pressure pump, which converted mechanical energy into pressure energy. The distribution system was mainly used to regulate the direction, speed and pressure of the liquid. The distribution system in this test was mainly utilized to regulate the pressure through the overflow valve. The working system mainly connected the liquid outlet to the test specimen, pressurized the test specimen, and converted the pressure energy into osmotic force. The working principal diagram of the pressure pump station and the pressure pump are shown in Figure 13 and Figure 14, respectively.

#### 2.2.3. Verification Test of Numerical Simulation

RFPA numerical calculation software was utilized to study the water pressure resistance of tunnel lining. The water pressure resistance of the lining (K2.0) in the transition section of karst cave and the lining (K3.0) in the section of karst cave of the New Yuanliangshan Tunnel were calculated and analyzed, and the results were compared with the large-scale model tests, so as to verify the reliability and accuracy of the model tests. It should be noted that the surrounding rock within 5 m around the karst cave with high water pressure was reinforced by grouting during the construction of the New Yuanliangshan Tunnel, so the influence of grouting stones was considered in the numerical calculation.

##### Establishment of Numerical Calculation Model

**Figure 15:**
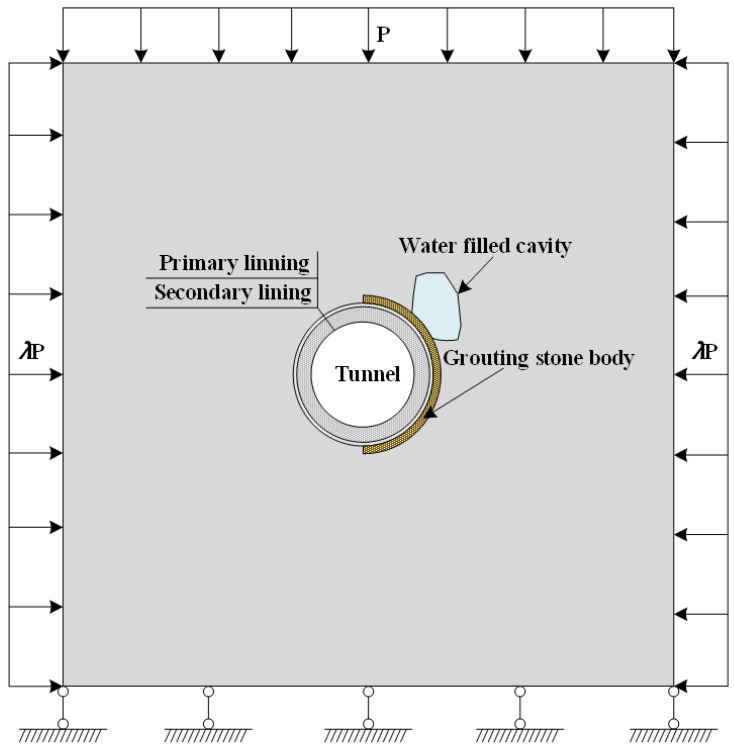
Schematic diagram of numerical calculation model.

In order to weaken the influence of boundary effect, the numerical model was taken as three times the tunnel diameter. The size of calculation model was 100 m × 100 m, and the tunnel inner diameter was 10 m. The thickness of grouting stone body was set as 5 m, and the thickness of primary lining was 0.2 m. In addition, the thickness of the secondary linings were 1.0 m and 1.2 m, respectively. Numerical calculation conditions were shown in Table 9. Water pressure was applied to the inner wall of the water-containing cave to simulate the effect of water pressure in cave. Initial water pressure of the cavity was 0.1 MPa, and the single step increment was 0.01 MPa. During numerical calculation, water pressure in cave was gradually increased until the lining structure broke and water inrush occurred. Numerical calculation model was shown in Figure 15. The numerical calculation was carried out by using a plane strain model, and the load-structure model was adopted as the numerical calculation model, which was divided into 400 × 400 = 160,000 units. A uniformly distributed load was applied at the upper, left and right boundary of the model, and vertical displacement constraint was applied at the lower boundary of the model. The buried depth of the New Yuanliangshan Tunnel was 500 m, and the average weight of surrounding rock was 20 kN/m^3^, so the uniform stress (p) on the upper boundary of the model was 10 MPa. Coefficient of lateral pressure (λ) was set to 1.5. Homogeneity of model (M) represented the homogeneity of material. The larger the value of M, the more homogeneous the macroscopic properties of material. Considering that the size of the smallest unit of the numerical calculation model was 25 cm, M could be set to 80. Since the compressive strength of rock is far greater than its tensile strength, the modified Mohr-Coulomb criterion considering tensile truncation was adopted as the strength criterion of unit failure.

##### Setting of Numerical Calculation Parameters

The rock and soil mass of the stratum of the karst cave section where the New Yuanliangshan Tunnel passed through was mainly limestone, and the filling material in the karst cave of the grouting stratum was mainly silty fine sand. According to the geological survey report and the test results of on-site borehole sampling, the physical and mechanical parameters of surrounding rock and grouting stone in the numerical calculation model were finally determined as shown in Table 10.

The primary lining of the tunnel was composed of reinforcement mesh + CF25 steel fiber shotcrete with a thickness of 20 cm + H200 steel frame, with a longitudinal interval of 50 cm. C30 concrete was adopted for the secondary lining. Numerical calculation parameters of support structure were shown in Table 11.

## 3. Results and Discussion

### 3.1. Results of Model Test

#### 3.1.1. Large-Scale Model Test of K2.0 Type Lining

**Figure 16:**
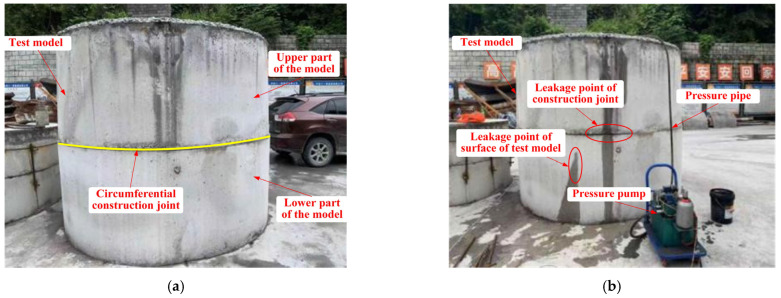
Loading process of model test: (**a**) test model before loading; (**b**) test model after loading.

The test process of water pressure resistance of the K2.0 type lining in the transition section of karst cave of the New Yuanliangshan Tunnel was shown in Figure 16. The pressurization of the model test was increased from 0 MPa to 7.0 MPa, with each increase of 0.5 MPa, and each level of pressurization was continuously stabilized for 30 min. The records of test process were shown in Table 12.

**Figure 17:**
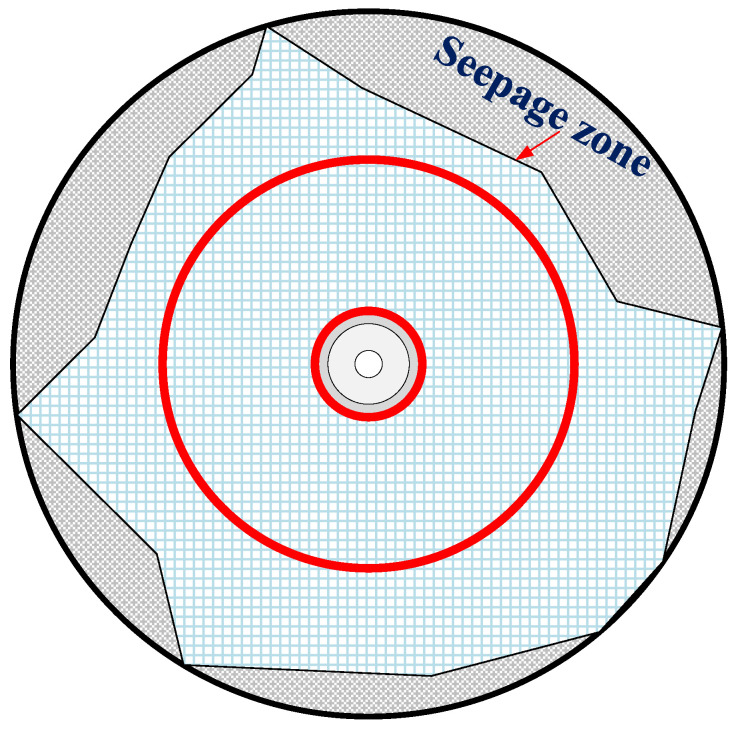
Water seepage path inside the test model.

Figure 17 and Table 12 showed the seepage path at the construction joint inside the test model. In the process of pressurization, it was found that the leakage range was gradually increased in the first 10 min, and then became stable in the last 20 min with each increase of 0.5 MPa. Leakage of the test model occurred when the pressure was increased to 5.0 MPa, which could be determined as the ultimate water pressure resistance of the model. Therefore, the water pressure resistance of K2.0 type lining obtained from the large-scale model test was 5.0 MPa. According to the conversion of safety factors, the water pressure resistance of the K2.0 type lining in the transition section of karst cave was 7.25 MPa when subjected to external uniform water pressure, and 3.33 MPa when subjected to external local water pressure, which met the design requirements of the New Yuanliangshan Tunnel.

#### 3.1.2. Large-Scale Model Test of K3.0 Type Lining

**Figure 18:**
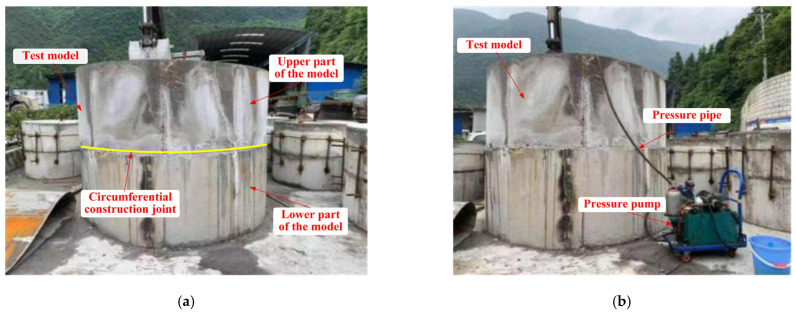
Loading process of model test: (**a**) test model before loading; (**b**) test model after loading (no leakage).

The test process of water pressure resistance of the K3.0 type lining in the section of karst cave of the New Yuanliangshan Tunnel was shown in Figure 18. The pressurization of the model test was increased from 0 MPa to 6.5 MPa, with each increase of 0.5 MPa, and each level of pressurization was continuously stabilized for 30 min. The records of test process were shown in Table 13.

Figure 18 and Table 13 showed that, in the process of pressurization, no leakage of the test model occurred when the pressure was increased to 6.5 MPa, which meant that the ultimate water pressure resistance of the model was not less than 6.5 MPa. Therefore, the water pressure resistance of K3.0 type lining obtained from the large-scale model test was not less than 6.5 MPa. According to the conversion of safety factors, the water pressure resistance of the K3.0 type lining in the section of karst cave was not less than 9.36 MPa when subjected to external uniform water pressure, and not less than 4.36 MPa when subjected to external local water pressure.

### 3.2. Verification Test Results of Numerical Calculation

The water pressure resistance of K2.0 and K3.0 type linings was studied by numerical simulation, the results of which were shown in Table 14. It could be seen from the numerical calculation results that the water pressure resistance values of the K2.0 type lining and K3.0 type lining were 3.56 MPa and 4.44 MPa, respectively.

**Figure 19:**
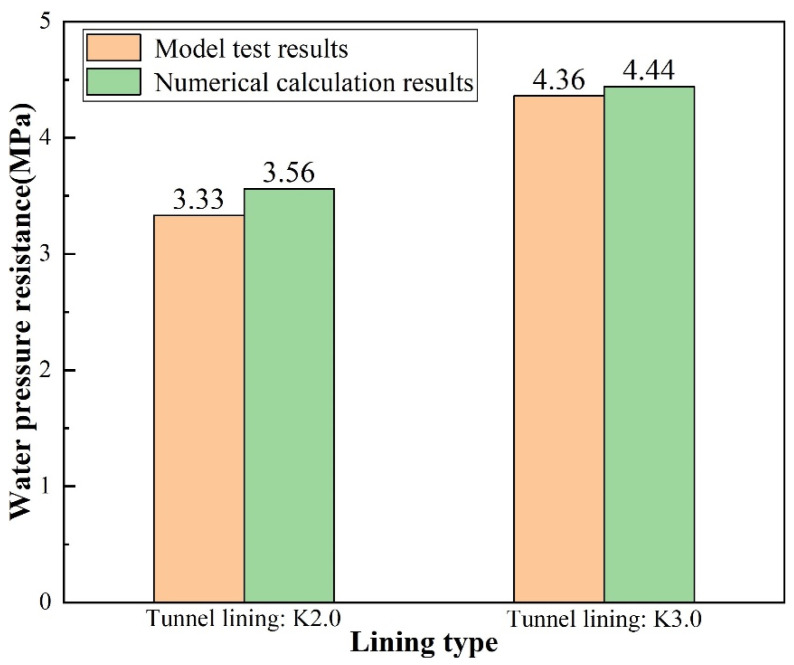
Histogram of water pressure resistance of lining structure obtained by model test and numerical calculation.

To sum up, the results of model test and numerical calculation were shown in Table 15 and plotted as a column chart, as shown in Figure 19.

Through comparative analysis of Table 15 and Figure 19, it could be seen that for K2.0 type lining, the results of model test and numerical calculation of its water pressure resistance were 3.33 MPa and 3.56 MPa, respectively, with a difference of only 6.4%. However, for K3.0 type lining, the model test result of the water pressure resistance of lining structure was not less than 4.36 MPa, while the numerical calculation result of that was 4.44 MPa, which was 1.8% higher than 4.36 MPa, thus verifying the accuracy of the large-scale model test. The research results indicated that the large-scale model test of water pressure resistance of tunnel lining had high reliability and could be extended to similar tunnel projects to test the ultimate water pressure resistance of lining structures.

## 4. Conclusions

The large-scale model test research on water pressure resistance of lining structure of high-pressure and water-rich tunnel was carried out in this paper, and the reliability of the model test was verified by using numerical calculation method. The main conclusions were as follows:

(1) For solving the problem of testing and verifying the water pressure resistance of lining structures of water-rich tunnels and the difficulty of implementing the existing model tests, the large-scale model test method that converted external water pressure of tunnel lining to internal water pressure for analyzing water pressure resistance of lining structures was creatively proposed. By utilizing this method, we verified that the lining structures of the New Yuanliangshan Tunnel crossing high-pressure and water-rich karst caves meet the design requirements of resisting water pressure of 3.0 MPa.
(2) The ultimate water pressure resistance of tunnel lining structure under external uniform water pressure and external local water pressure was 1.44 times and 0.67 times of that obtained from the large-scale model tests, respectively. The results of large-scale model tests indicated that the water pressure resistance of the K2.0 type lining in the transition section of karst cave and the K3.0 type lining in the section of karst cave of the New Yuanliangshan Tunnel was 3.33 MPa and no less than 4.36 MPa, respectively. Additionally, the accuracy of the large-scale model tests was verified by numerical calculation.
(3) The large-scale model test method for testing and verifying water pressure resistance of tunnel lining structure provided a new idea and reference for the research on water pressure resistance of tunnel lining. In addition, the model test method reduced the difficulty of testing the water pressure resistance of tunnel lining, and the numerical calculation verified its high reliability and accuracy, which could be extended to similar tunnel projects and other types of tunnel lining under the action of water pressure to study the water pressure resistance of lining structures.

## Figures and Tables

**Table 1:** Concrete mixture for large-scale model test.

Concrete Mixtures	Properties	Standards Conformed To
Cement	P.O Type 42.5 Portland cement; Density: 3100 kg/m^3^	Common Portland Cement (China National Standard GB 175-2007)
Water	Potable water	Standards for Drinking Water Quality (China National Standard GB 5749-2022)
Coarse aggregates	5~31.5 mm continuous graded crushed gravel; Specific gravity: 2.56	Pebble and Crushed Stone for Construction (China National Standard GB/T 14685-2011)
Fine aggregates	0.2~5 mm natural river sand; Specific gravity: 2.62; Fineness modulus: 2.59	Sand for Construction (China National Standard GB/T 14684-2011)
Admixture	High range water reducer (HRWR); Water reduction rate: 20%~35%; Solid content: 20%	Concrete Admixtures (China National Standard GB 8076-2008)

**Table 2:** Physical and mechanical properties of Portland cement.

Initial Setting Time	Final Setting Time	Stability of Cement	Compressive Strength (MPa)		Bending Strength (MPa)	
			3 Days	28 Days	3 Days	28 Days
150 min	200 min	Qualified	26.6	47.7	4.7	7.6

**Table 3:** The quality test of potable water.

Test Item	Test Result	Regulatory Standard (GB 5749-2022)
Total dissolved solids (mg/L)	405	<1000
Chlorine (mg/L)	94	250
Fe (mg/L)	0.020	<0.3
Al (mg/L)	0.0020	<0.2
Mn (mg/L)	0.0031	<0.1
pH value	7.36	6.5~8.5

**Table 4:** Mix identifications and ratios of cubic specimens.

Water (g)	Cement (g)	Aggregate (g)		HRWR (g)
		Fine	Coarse	
760	1440	2680	4480	10.08

**Table 5:** Numerical calculation conditions.

Calculation Condition	I	II	III	IV
Loading mode	Lining bearing external uniform water pressure	Lining bearing external local water pressure	Lining bearing internal uniform water pressure	Large-scale test model bearing internal uniform water pressure

**Table 6:** Numerical calculation parameters.

Parameter Type	Uniaxial Compressive Strength (MPa)	Elastic Modulus (GPa)	Poisson’s Ratio	Ratio of Compression Strength to Tensile Strength	Heterogeneity
Parameter value	30	30.9	0.23	10	100

**Table 7:** Safety factor of water pressure resistance.

Calculation Condition	I	II	III	IV
Loading mode	Lining bearing external uniform water pressure	Lining bearing external local water pressure	Lining bearing internal uniform water pressure	Large-scale test model bearing internal uniform water pressure
Water pressure resistance (MPa)	9.1	4.2	3.0	6.3
Safety factor	3.03	1.4	1.0	2.1

**Table 8:** Application condition and waterstop setting of the large-scale test models.

Specimen No	Applicable Section of Model	Setting of Waterstop
1	Transition section of karst cave	Circumferential construction joint: type II construction joint; Layout form of waterstop: back stick rubber waterstop + mid-buried steel plate waterstop
2	Section of karst cave	Circumferential construction joint: type III construction joint; Layout form of waterstop: back stick rubber waterstop + mid-buried steel plate waterstop + mid-buried corrugated steel waterstop

**Table 9:** Numerical calculation conditions.

Thickness of Primary Lining (m)	Thickness of Secondary Lining (m)	Thickness of Grouting Stone (m)
0.2	1.0; 1.2	5.0

**Table 10:** Numerical calculation parameters of surrounding rock and grouted stone.

Parameter type	Elastic Modulus (GPa)	Poisson’s Ratio	Angle of Internal Friction (°)	Permeability Coefficient (m/d)	Cohesion: C (Mpa)	Bulk Density: *γ* (kN/m^3^)
Surrounding rock	0.48	0.3	35	0.044	0.05	20
Grouting stone	2.0	0.35	35	0.00443	0.065	24

**Table 11:** Numerical calculation parameters of support structure.

Support Structure	Density (Kg/m^3^)	Elastic Modulus (GPa)	Poisson’s Ratio
Primary lining	2481.3	28.7	0.23
Secondary lining	2386.8	30.9	0.23

**Table 12:** Records of test process.

Number	Pressure (MPa)	Stabilization Time of Pressurization (Minute)	Leakage
1	0.5	30	No leakage point
2	1.0	30	No leakage point
3	1.5	30	No leakage point
4	2.0	30	No leakage point
5	2.5	30	No leakage point
6	3.0	30	No leakage point
7	3.5	30	No leakage point
8	4.0	30	No leakage point
9	4.5	30	No leakage point
10	5.0	30	One leakage point appeared
11	5.5	30	Leakage occurred on the surface of test model
12	6.0	30	Leakage occurred at the bottom of test model, and the leakage speed was accelerated
13	6.5	30	The leakage speed was accelerated
14	7.0	30	Leakage remained stable

**Table 13:** Records of test process.

Number	Pressure (MPa)	Stabilization Time of Pressurization (Minute)	Leakage
1	0.5	30	No leakage point
2	1.0	30	No leakage point
3	1.5	30	No leakage point
4	2.0	30	No leakage point
5	2.5	30	No leakage point
6	3.0	30	No leakage point
7	3.5	30	No leakage point
8	4.0	30	No leakage point
9	4.5	30	No leakage point
10	5.0	30	No leakage point
11	5.5	30	No leakage point
12	6.0	30	No leakage point
13	6.5	30	No leakage point

**Table 14:** Numerical calculation results of water pressure resistance of lining structure.

Lining Type	K2.0 (Thickness: 1.0 m)	K3.0 (Thickness: 1.2 m)
Water pressure resistance (MPa)	3.56	4.44

**Table 15:** Results of model test and numerical calculation of water pressure resistance of lining structure.

Lining Type	K2.0 (Thickness: 1.0 m)	K3.0 (Thickness: 1.2 m)
Numerical calculation results of water pressure resistance (MPa)	3.56	4.44
Model test results of water pressure resistance (MPa)	3.33	≥4.36

## Data Availability

Not applicable.
